# Neurologie in der DDR: eine systematische Literaturübersicht zur historischen Aufarbeitung

**DOI:** 10.1007/s00115-022-01354-7

**Published:** 2022-07-12

**Authors:** Jan Armbruster, Matthias Grothe, Kathleen Haack, Ekkehardt Kumbier

**Affiliations:** 1 Klinik für Forensische Psychiatrie und Psychotherapie, Helios Hanseklinikum Stralsund, Stralsund, Deutschland; 2 Klinik und Poliklinik für Neurologie, Universitätsmedizin Greifswald, Greifswald, Deutschland; 3 Klinik und Poliklinik für Psychiatrie und Psychotherapie, Universitätsmedizin Greifswald, Greifswald, Deutschland; 4 Arbeitsbereich Geschichte der Medizin, Universitätsmedizin Rostock, Doberaner Str. 140, 18057 Rostock, Deutschland

**Keywords:** Historische Aufarbeitung, DDR, Geschichte, Fächerdifferenzierung, Systematisches Review, Historical reappraisal, East Germany, History, Medical specialization, Systematic review

## Abstract

**Hintergrund:**

Die Entwicklung der Neurologie verlief in beiden deutschen Staaten nach 1945 unterschiedlich, wobei nur wenig über die Neurologie in der DDR bekannt ist.

**Fragestellung:**

Der Stand der historischen Forschung zur Neurologie in der DDR soll aufgezeigt werden.

**Material und Methode:**

Es erfolgte eine systematische Literaturrecherche für den Zeitraum 1991 bis 2021 sowie eine Einordnung der jeweiligen Beiträge in verschiedene Kategorien.

**Ergebnisse:**

Neben einer regional sehr unterschiedlichen Aufarbeitung zu spezifischen Themen zeigt sich insgesamt ein Mangel an einer thematischen Gesamtdarstellung sowie an Arbeiten zu gesellschaftspolitischen Zusammenhängen innerhalb der DDR und vergleichenden Aspekten im deutsch-deutschen, aber auch internationalen Maßstab.

**Schlussfolgerungen:**

Die systematische Forschung der Geschichte der Neurologie in der DDR unter Berücksichtigung der Rolle innerhalb des sozialistischen Gesundheitswesens sollte im Rahmen eines separaten Forschungsprojekts aufgearbeitet werden und dabei vergleichende Aspekte einbeziehen.

## Abstract (en)

**Background:**

Neurology as a discipline developed differently in the two German states after 1945 and little is known about neurology in the GDR.

**Objective:**

This article examines the present state of historical research on neurology in the GDR.

**Materials and methods:**

We systematically screened the existing literature from the period 1991–2021 and assigned the studies to different categories.

**Results:**

We found significant regional differences in the way specific topics are addressed. We also identified a lack of a general overview on the subject and a lack of studies dealing with neurology within the specific socio-political context of the GDR. Comparative studies between the GDR and the BRD and between the GDR and other countries are also scarce.

**Conclusion:**

Further systematic research (in the form of a separate research project) into the history of neurology in the GDR and its role within the socialist healthcare system of the GDR is needed, taking comparative aspects into account.

Die Entwicklung der Neurologie im geteilten Deutschland verlief nach 1945 äußerst divergent: Während sich in der Bundesrepublik Deutschland (BRD) spätestens ab Anfang der 1960er-Jahre klare Autonomiebestrebungen und eine zunehmende Abgrenzung gegenüber der Psychiatrie zeigen, blieben in der Deutschen Demokratischen Republik (DDR) beide Fächer, nicht zuletzt aus ideologischen Gründen, eng miteinander verbunden [14]. Erst Anfang der 1980er-Jahre führte die zunehmende Profilierung und Spezialisierung der Neurologie zur Sektionsbildung in der Fachgesellschaft für Psychiatrie und Neurologie und so zur Emanzipation des Fachs [18]. Eigenständige Kliniken etablierten sich zumeist erst nach 1990 [14].

Diese Entwicklungsprozesse sind im Gegensatz zur Psychiatrie mit laufenden Projekten zur Psychiatriehistoriografie in der DDR [23], abgeschlossenem Forschungsauftrag der Deutschen Gesellschaft für Psychiatrie und Psychotherapie, Psychosomatik und Nervenheilkunde (DGPPN) [13] sowie einer Reihe von Publikationen [28, 34, 43, 44] bisher kaum erforscht. Die vorliegende Arbeit gibt einen Überblick über den aktuellen Forschungsstand zur Neurologie in der DDR und möchte damit die historisch-kritische Aufarbeitung des Fachgebiets anregen.

## Methoden

**Figure 1:**
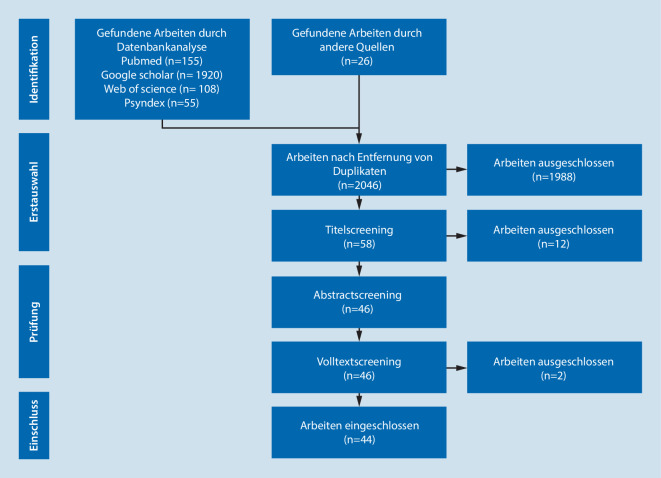

Es erfolgte eine systematische Literaturrecherche (Abb. 1) in den Datenbanken Pubmed, Web of Science und Psyndex und der Suchmaschine Google Scholar, um sowohl Zeitschriftenartikel als auch Buchbeiträge für den Zeitraum 1991 bis 2021 zu detektieren.

Die Suchstrategie umfasste die Begriffe neurolog* AND GDR, neurolog* AND east germany, neurophysiolog* AND GDR, neurophysiolog* AND east germany, neuropsycholog* AND GDR und neuropsycholog* AND east germany. In Psyndex als deutschsprachiger Datenbank wurde analog nach den deutschen Begriffen gesucht. Nach Ausschluss von Duplikaten, unabhängigem Titelscreening und nachfolgender Auswertung wurden Titel eingeschlossen, die von mindestens zwei Autoren ausgewählt worden waren. Da sich zeigte, dass einige den Autoren geläufige Beiträge nicht erfasst werden konnten, wurden zusätzlich alle bekannten und nicht erfassten Titel hinzugefügt (Abb. 1 andere Quellen) und dem weiteren Screeningprozess unterzogen. Die ausgewählten Beiträge wurden folgenden Kategorien zugeordnet; Mehrfacheinordnungen waren möglich:

1. Institutionen
2. Personen
3. Diagnostik und Therapie
4. Fächerdifferenzierung
5. Fachgesellschaften und Fachzeitschriften
6. Politische und ideologische Einflussnahme

Für die Zuordnung war ein unabhängiges Rating durch mehr als zwei Autoren und die Bestätigung in der nachfolgenden Diskussion erforderlich.

## Ergebnisse

Es wurden 44 Publikationen eingeschlossen (Tab. 1):

**Table 1:** 

Autoren	Jahr	Kategorien	Rubrik	Inhalt
Schmiedebach [42]	1999	Institutionen	Buchbeitrag	Entwicklung der Neurologie in Greifswald von den Anfängen bis 1974
		Fächerdifferenzierung		
Thomas [49]	2002	Fachgesellschaften und Fachzeitschriften	Dissertation	Zusammenfassung der Fachgesellschaft für Neurologie und Psychiatrie im Vergleich zwischen Ost- und Westberlin
Sayk [41]	2003	Personen	Buch	Autobiografie des Neurologen Johannes Sayk
Wagner [50]	2007	Fächerdifferenzierung	Buchbeitrag	Allgemeine Entwicklung der Neurologie in der DDR
		Fachgesellschaften und Fachzeitschriften		
Wagner [51]	2007	Institutionen	Buchbeitrag	Profilbildung der Neurologie in der DDR
		Fächerdifferenzierung		
Eisenberg [14]	2007	Fächerdifferenzierung	Buchbeitrag	Allgemeine Beschreibung der Neurologie in DDR und BRD
		Fachgesellschaften und Fachzeitschriften		
Gerhard et al. [19]	2007	Personen	Zeitschriftenartikel	Kurzbiografie Rudolf Lemke mit Fokus auf Entwicklung der Kinderneuropsychiatrie in Jena
		Institutionen		
		Fächerdifferenzierung		
Kumbier et al. [32]	2009	Institutionen	Zeitschriftenartikel	Entstehung des ersten eigenständigen neurologischen Lehrstuhls in der DDR in Rostock (1958)
		Fächerdifferenzierung		
		Politische und ideologische Einflussnahme		
Kumbier et al. [31]	2009	Personen	Zeitschriftenartikel	Politisch intendierte Entlassung des Direktors der Universitäts-Nervenklinik Rostock 1958
		Institutionen		
		Fächerdifferenzierung		
		Politische und ideologische Einflussnahme		
Kumbier et al. [35]	2009	Personen	Englischsprachiger Zeitschriftenartikel	Kurzvorstellung Johannes Sayk als Pionier der Neurologie
		Diagnostik und Therapie		
Kumbier [24]	2009	Fachgesellschaften und Fachzeitschriften	Buchbeitrag	Übersicht der Entwicklung der regionalen und nationalen Fachgesellschaft(en) für Psychiatrie und Neurologie in der DDR
		Politische und ideologische Einflussnahme		
Armbruster [1]	2010	Personen	Buchbeitrag	Biografie Kurt Erich Moser als Gründer der neurologischen Klinik Stralsund
Eisenberg [15]	2010	Politische und ideologische Einflussnahme	Buchbeitrag	Beschreibung des Pawlowismus als ideologisches Konzept in der Neurologie
Kumbier et al. [33]	2010	Personen	Zeitschriftenartikel	Entwicklung der Kinderneuropsychiatrie in Rostock
		Institutionen		
		Fächerdifferenzierung		
Kumbier [25]	2010	Personen	Habilitationsschrift	Entwicklung der Nervenheilkunde in der DDR zwischen 1946 und 1961
		Politische und ideologische Einflussnahme		
Boide et al. [9]	2011	Diagnostik und Therapie	Zeitschriftenartikel	„Zentralstelle für Morbus Wilson“ der DDR in Leipzig
Eisenberg [16]	2011	Fachgesellschaften und Fachzeitschriften	Buchbeitrag	Übersicht über den persönlichen und inhaltlichen Austausch der west- und ostdeutschen Fachgesellschaften für Neurologie zwischen 1945 und 1970
		Politische und ideologische Einflussnahme		
Eisenberg [17]	2012	Fachgesellschaften und Fachzeitschriften	Buchbeitrag	Kontroverse um die Aufnahme der DDR in die World Federation of Neurology (1960)
		Politische und ideologische Einflussnahme		
Matthies [36]	2012	Personen	Buch	Autobiografie inklusive der Entstehungsgeschichte des Zentrums für Neurowissenschaften in Magdeburg
		Fächerdifferenzierung		
Teitge [46]	2013	Fachgesellschaften und Fachzeitschriften	Dissertationsschrift	Entstehung der einzigen DDR-Fachzeitschrift für Neurologie und Psychiatrie und Analyse der Fachbeiträge
		Politische und ideologische Einflussnahme		
Kumbier et al. [30]	2015	Personen	Zeitschriftenartikel	Hochschullehrer an den Universitäts-Nervenkliniken in der SBZ und DDR bis 1961
		Institutionen		
		Politische und ideologische Einflussnahme		
Teitge et al. [47]	2015	Fachgesellschaften und Fachzeitschriften	Zeitschriftenartikel	Siehe Teitge [46]
		Politische und ideologische Einflussnahme		
Wagner et al. [58]	2015	–	Buch	Entwicklung der Neurologie an der Universität Leipzig
a) Wagner et al. [55]		Personen	Buchbeitrag	Universitäts-Nervenklinik Leipzig von 1945 bis 1952
		Institutionen		
		Diagnostik und Therapie		
		Fächerdifferenzierung		
b) Wagner et al. [56]		Personen		Universitäts-Nervenklinik Leipzig von 1953 bis 1964/65
		Institutionen		
c) Wagner et al. [57]		Personen		Neurologische Abteilung der Universitäts-Nervenklinik Leipzig von 1945 bis 1985
		Institutionen		
		Fächerdifferenzierung		
d) Wagner [52]		Institutionen		Abteilungen der Universitäts-Nervenklinik Leipzig von 1965 bis 1976
e) Wagner [53]		Personen		Neurologische Abteilung inklusive Personen und Tätigkeiten der Universitäts-Nervenklinik Leipzig von 1965 bis 1985
		Institutionen		
		Diagnostik und Therapie		
		Fächerdifferenzierung		
f) Wagner [54]		Institutionen		Neurologische Abteilung inklusive Schwerpunktbildung der Universitäts-Nervenklinik Leipzig von 1945 bis 1985
		Fächerdifferenzierung		
Teitge et al. [48]	2015	Fachgesellschaften und Fachzeitschriften	Zeitschriftenartikel	Entwicklung der einzigen DDR-Fachzeitschrift
		Politische und ideologische Einflussnahme		
Kumbier [26]	2016	Fachgesellschaften und Fachzeitschriften	Zeitschriftenartikel	Entstehung der Gesellschaft für Psychiatrie und Neurologie in Mecklenburg in der SBZ
		Politische und ideologische Einflussnahme		
Reiber [39]	2016	Diagnostik und Therapie	Zeitschriftenartikel	Überblick über die Liquordiagnostik in der BRD und DDR
Häßler [20]	2016	Personen	Zeitschriftenartikel	Entstehung der Kinderneuropsychiatrie inklusive des ersten Lehrstuhls in der DDR in Rostock (1958)
		Fächerdifferenzierung		
Bashian [7]	2016	Personen	Dissertationsschrift	Leben und Werk von Johannes Sayk
		Diagnostik und Therapie		
Dahlmann et al. [11]	2017	Personen	Englischsprachiger Zeitschriftenartikel	Zellsedimentationskammer nach Sayk und deren historische Einordnung hinsichtlich der Liquordiagnostik
		Diagnostik und Therapie		
Häßler [21]	2017	Personen	Buchbeitrag	Kinder- und Jugendneuropsychiatrie in der DDR
		Fächerdifferenzierung		
Bart et al. [5]	2018	Personen	Zeitschriftenartikel	Biografie Bernhard Schwarz und seine Untersuchungen von Boxern hinsichtlich neurodegenerativer Erkrankungen
		Diagnostik und Therapie		
		Fächerdifferenzierung		
Bart et al. [6]	2018	Personen	Zeitschriftenartikel	Siehe Bart et al. [5]
		Diagnostik und Therapie		
		Fächerdifferenzierung		
Bashian et al. [8]	2018	Personen	Buchbeitrag	Leben und Wirken von Johannes Sayk
		Diagnostik und Therapie		
Kumbier [27]	2019	Personen	Buchbeitrag	Fächerdifferenzierung an der Universität Rostock mit Entstehung des ersten neurologischen Lehrstuhls (1958) in der DDR
		Institutionen		
		Fächerdifferenzierung		
		Politische und ideologische Einflussnahme		
Strassburg [45]	2020	Fachgesellschaften und Fachzeitschriften	Englischsprachiger Zeitschriftenartikel	Entwicklung der westdeutschen Gesellschaft für Neuropädiatrie mit der eingeschränkten Möglichkeit des Austausches mit Fachvertretern in der DDR
		Fächerdifferenzierung		
Polianski [37]	2020	Politische und ideologische Einflussnahme	Zeitschriftenartikel	Politische Einflussnahme auf Fachbücher in der SBZ und frühen DDR
Rzesnitzek et al. [40]	2020	Fächerdifferenzierung	Englischsprachiger Zeitschriftenartikel	Entwicklung der stereotaktischen Neurochirurgie in Europa inklusive BRD und DDR
Kumbier [29]	2020	Fachgesellschaften und Fachzeitschriften	Buchbeitrag	Gründung der Fachgesellschaften für Psychiatrie und Neurologie in der DDR
		Politische und ideologische Einflussnahme		
Armbruster [2]	2020	Institutionen	Buchbeitrag	Fächerdifferenzierung von Neurologie und Psychiatrie an der Universität Greifswald
		Fächerdifferenzierung		
		Politische und ideologische Einflussnahme		
Armbruster [3]	2020	Institutionen	Buchbeitrag	Überblick über die Geschichte der Neurologie in Greifswald
		Fächerdifferenzierung		
Brandhoff et al. [10]	2020	Personen	Zeitschriftenartikel	Nachruf auf Detlef Müller
Rabending et al. [38]	2021	Diagnostik und Therapie	Zeitschriftenartikel	Überblick über die Epileptologie in der DDR
		Fächerdifferenzierung		
Armbruster [4]	2021	Personen	Buchbeitrag	Leben und Werk von Karl-Heinz Elsaesser
		Institutionen		
		Politische und ideologische Einflussnahme		
Dörre [12]	2021	Fachgesellschaften und Fachzeitschriften	Buchkapitel	Zuständigkeitskonflikte der Fachgesellschaften für Neurologie und Psychiatrie in der DDR
		Fächerdifferenzierung		

*BRD* Bundesrepublik Deutschland, *DDR* Deutsche Demokratische Republik, *SBZ* Sowjetische Besatzungszone

## Diskussion

Die systematische Literaturrecherche für den 30-jährigen Zeitraum ergab mit 44 Beiträgen eine geringe Zahl von Veröffentlichungen zur Neurologie in der DDR, die sich inhaltlich zudem teilweise stark überschneiden. Damit wurde die Vorannahme bestätigt, dass ein nationaler sowie vergleichend internationaler medizinhistorischer Forschungsbedarf besteht. Zunächst zeigt sich, dass das Thema erst verstärkt ab 1999 aufgegriffen wurde, was vermutlich zum einen an dem zeitlichen Abstand, der zur Historisierung und damit differenzierten Geschichtsschreibung notwendig ist, zum anderen an der Verfügbarkeit von Archivalien, die aufgrund von Schutzbestimmungen erst in jüngster Zeit zugänglich geworden sind, liegt.

Die 17 Arbeiten zu Institutionen weisen abgesehen von zwei Übersichtsarbeiten zu verschiedenen Einrichtungen [30, 51] die regionalen Schwerpunkte Rostock (5) [4, 27, 31–33], Greifswald (4) [2–4, 42] sowie Leipzig (6 Kapitel [52–57] in einer Buchpublikation [58]) auf. In Rostock finden sich mit der Schaffung des ersten separaten neurologischen Lehrstuhls (1958; [32]) und in Leipzig mit der Einrichtung der DDR-weit einzigen rein neurologischen Universitätsklinik (1965; [53, 57]) Hinweise für eine beginnende frühe Eigenständigkeit, die auch zu einer schärferen fachlichen Abgrenzung gegenüber der Psychiatrie führte. In Greifswald hingegen bestand durchgängig eine verbundene Klinik [2]. Die regionale Aufarbeitung kann durch das wissenschaftshistorische Interesse der jeweiligen Hochschuleinrichtungen erklärt werden, was sich auch auf die Kategorien Personen, Diagnostik und Therapie sowie Fächerdifferenzierung auswirkt.

Bei den 23 Beiträgen zu einzelnen Personen finden sich neben Arbeiten zu mehreren Personen [25, 30, 36] eine systematische Darstellung der neurologischen Fachvertreter der Universität Leipzig bis 1985 [53, 55–57] und neun Veröffentlichungen zu den Rostocker Professoren Franz Günther von Stockert [27, 31], Gerhard Göllnitz [20, 21, 33] und Johannes Sayk [7, 8, 11, 35, 41]. Die intensive Beschäftigung mit Johannes Sayk basiert auf der Bedeutung seiner Forschungen zur Liquorzytologie und deren wissenschaftlicher Anerkennung auf nationaler und internationaler Ebene. Bei Franz Günther von Stockert spielt die politische Brisanz, bei Gerhard Göllnitz die Begründung der Kinderneuropsychiatrie im Rahmen der Fächerdifferenzierung eine Rolle. Darüber hinaus fanden sich Beiträge zu Bernhard Schwarz [5, 6], Rudolf Lemke [19], Kurt Moser [1], Detlef Müller [10] und Karl-Heinz Elsaesser [4].

Auch bei den 11 Aufsätzen der Kategorie Diagnostik und Therapie beschäftigen sich allein fünf Arbeiten mit der Liquordiagnostik [7, 8, 11, 35, 39], bei den Beiträgen zur Epileptologie [38] und der Neurotraumatologie [5, 6] liegt der Fokus auf der Subspezialisierung. Dazu fand sich für Leipzig mit der Zentralstelle für Morbus Wilson ein Alleinstellungsmerkmal [9, 53].

Von insgesamt 24 Veröffentlichungen der Kategorie Fächerdifferenzierung fokussiert eine ganze Reihe auf Abgrenzungstendenzen zwischen Neurologie und Psychiatrie [2, 3, 12, 27, 32, 42, 55, 57]. Neben der Differenzierung der Kinderneuropsychiatrie bzw. Neuropädiatrie [19–21, 33, 45] beschäftigen sich einzelne Arbeiten mit speziellen Subspezialisierungen wie der Epileptologie [38], der Abgrenzung zur Neurotraumatologie [5, 6] oder Neurochirurgie [40].

In der Kategorie Fachgesellschaften und Fachzeitschriften finden sich Arbeiten über die einzige Fachzeitschrift, die zugleich als Mitteilungsorgan der Gesellschaft für Psychiatrie und Neurologie der DDR fungierte, wodurch von einer engen Verzahnung zwischen der Fachgesellschaft und der Schriftleitung der Zeitschrift ausgegangen werden muss. Die Fachzeitschrift wurde über den gesamten Zeitraum des Bestehens der DDR untersucht [46], deren Beiträge analysiert [48] und ihre Entstehungsgeschichte im gesellschaftspolitischen Kontext aufgearbeitet, wobei bisher auf die psychiatrischen und weniger auf die neurologischen Inhalte eingegangen wurde [47]. In den insgesamt 13 Beiträgen werden darüber hinaus verschiedene Teilaspekte der Gründungsgeschichte der Fachgesellschaft, ihre Fortentwicklung mit Bildung einer eigenständigen Sektion Neurologie [24, 29, 50], Konflikte in den Überschneidungsbereichen zur Psychiatrie und weiteren Bereichen [12], Unterschiede und Gemeinsamkeiten zwischen beiden deutschen Fachgesellschaften [14, 16, 17] wie auch die Entwicklung von Regionalgesellschaften [26], u. a. im Vergleich der Ost- mit der Westberliner Fachgesellschaft [49], erörtert.

In 17 Arbeiten spielen politische Aspekte eine wichtige Rolle. Mit der zunehmenden politisch-ideologischen Einflussnahme der SED-Verantwortlichen (Sozialistische Einheitspartei Deutschlands) wurden die gesellschaftlichen, wissenschaftlichen und legislativen Rahmenbedingungen für zentralistisch organisierte Strukturen im Gesundheits- [4, 24–26, 29] und Hochschulwesen mit der entsprechenden Kaderpolitik [2, 4, 25, 27, 30–32] geschaffen. Als Mittel der Beeinflussung finden sich Zensur sowie das ideologiegeleitete Präferieren wissenschaftlicher Inhalte [37, 46–48]. Ein wichtiger Aspekt der ideologischen Einflussnahme zeigt sich in dem auch spezifisch auf die Neurologie zielenden [16] Versuch der Etablierung des „Pawlowismus“, der in der DDR vor allem in den 1950er-Jahren propagiert wurde und als Versuch zu verstehen ist, die Forschung stärker am Vorbild der sowjetischen Wissenschaften zu orientieren [15]. Unter den Arbeiten, die sich mit der Förderung bzw. Verhinderung eines Wissenschaftsaustauschs auf nationaler und internationaler Ebene auseinandersetzen [16, 17, 26, 46, 48], sticht eine durch einen Perspektivwechsel heraus, indem sie das Bestreben politisch Verantwortlicher in der BRD untersucht, die Aufnahme der DDR-Gesellschaft in die World Federation of Neurology zu unterbinden und die mit einer solchen Mitgliedschaft verbundene internationale Anerkennung zu erschweren [17].

Dass die bei der Literaturrecherche identifizierten Publikationen bis auf drei englischsprachige Arbeiten [11, 35, 45] in deutscher Sprache veröffentlicht wurden, unterstreicht das bisher eher nationale Interesse an der historischen Forschung zur Neurologie in der DDR. Vergleichende Einordnungen zu Entwicklungen in der BRD, auf internationaler Ebene und insbesondere gegenüber anderen sozialistischen Staaten fehlen weitgehend. Systematische Untersuchungen, etwa analog dem Forschungsprojekt „Neurologie in der NS-Zeit“ der Deutschen Gesellschaft für Neurologie (DGN) [22], fehlen vollständig.

Methodenkritisch ist anzumerken, dass die initial gewählte systematische Recherche Schwächen bei der umfassenden Identifikation der Veröffentlichungen aufwies, was eine Anpassung über die Ergänzung mit anderen Quellen erforderte. Dabei ist nicht auszuschließen, dass einzelne Beiträge nicht erfasst wurden, was die Gesamtaussage jedoch nur wenig beeinflussen dürfte.

## Fazit für die Praxis

Auch wenn einige Arbeiten zu verschiedenen Teilaspekten vorliegen, fehlt bisher die spezifische historische Aufarbeitung des Themas Neurologie in der DDR. Für eine systematische Herangehensweise sollten neben der gezielten Archivarbeit und der Analyse der Primärliteratur auch Zeitzeugen interviewt werden. Inhaltlich erscheinen Themen wie der Bezug zum DDR-Gesundheitssystem und zu den Strukturen des SED-Staates, lokale und regionale inhaltliche Schwerpunktbildungen sowie der nationale und internationale Wissenschaftsaustausch lohnende und spannende Forschungsfelder.
